# Reply to Newton: Missing the forest (correlation) for the trees (mean differences)

**DOI:** 10.1073/pnas.2314007120

**Published:** 2023-10-03

**Authors:** Jason C. K. Chan, Dahwi Ahn

**Affiliations:** ^a^Department of Psychology, Iowa State University, Ames, IA 50014

In Chan and Ahn (1), we argued that unproctored online exams can accurately assess learning. As a secondary discovery, we also suggested that our data either showed little evidence of widespread cheating or that “perhaps more likely, …cheating was ineffective at boosting performance…” We also considered the “additional possibility that practically everyone cheats during online exams.” Nonetheless, Newton (2) incorrectly stated that we had claimed that cheating was unlikely in online exams. But perhaps more importantly, Newton focused on the average difference between online and in-person exam scores and argued this difference as evidence of cheating. As we have stated in our paper, an overall increase in test scores for online exams can be readily remediated (by changing grade cutoffs or making the exams more difficult). What is more important is whether online and in-person exams assess learning in a similar manner, and the evidence unequivocally shows that they did (*r* = 0.59).

But let us also consider Newton’s assertion that the grade inflation associated with online exams is evidence of cheating. Although we acknowledge that the incidence of cheating might be higher in online exams—despite some other recent evidence to the contrary (3)—the grade inflation for online exams in our data might not suggest cheating (4). One possibility we entertained was that instructors were more likely to use multiple-choice questions in online exams. Newton rightly noted that we did not provide evidence for this argument, so we have re-examined our data (and provided these updated data on our OSF page). We found that five courses either eliminated open-ended questions or substantially increased the proportion of multiple-choice questions for online exams, two additional courses (*N* = 299) officially adopted an open-notes policy for online exams, and eight courses (*N* = 678) provided students with an extended exam window (e.g., 12 to 168 h). Together, these factors could account for a substantial proportion of the grade inflation (5, 6), which was about 5% in exam scores (*g* = 0.40).

Even if we concede that cheating might have partially improved students’ scores, our data still showed a powerful correlation between their scores on the in-person and online exams. Again, group-level (average) differences between in-person and online exams are far less informative than the individual-level correspondence (i.e., correlation) between the scores that students achieved in online and in-person exams. There would be a cause for concern if grade inflation were so pronounced that nearly all students performed at a similarly high level in online exams (e.g., 90%). However, this clearly did not happen. Instead, we found that online exams differentiated student performance just as in-person exams did.

Finally, we agree with Newton that the rise of ChatGPT and similar tools might increase the prevalence of cheating. But how these technologies might impact the ability of unproctored online exams to assess learning is still unknown. It seems premature to conclude that “unproctored online exams are not meaningful” and “should be avoided,” as Newton did.
